# Editorial: Enhancing the health of vulnerable populations through integrated biosocial approaches

**DOI:** 10.3389/fmed.2026.1910231

**Published:** 2026-09-11

**Authors:** Echu Liu, Yiu Wing Kam, Huilin Cheng

**Affiliations:** 1 Department of Health Management and Policy, College for Public Health and Social Justice, Saint Louis University, Saint Louis, MO, United States; 2 Division of Natural and Applied Sciences, Duke Kunshan University, Suzhou, Jiangsu, China; 3 School of Nursing, Hong Kong Polytechnic University, Kowloon, Hong Kong SAR, China

**Keywords:** health equity, healthcare professionals, healthcare systems, integrated biosocial approaches, patient-centered care, vulnerable populations

Contemporary medicine increasingly encounters forms of vulnerability that cannot be understood through disease processes alone. Biological susceptibility intersects with psychological well-being, behavior, social conditions, life-stage transitions, healthcare access, and the organization of care. An integrated biosocial perspective therefore requires more than identifying which populations are vulnerable; it requires understanding how complex needs become clinically recognizable and how healthcare professionals and systems can respond to them. The Research Topic “Enhancing the Health of Vulnerable Populations Through Integrated Biosocial Approaches” brings together studies spanning biological mechanisms, clinical care, professional practice, and healthcare systems. Read together, these contributions identify three linked points at which vulnerability becomes clinically actionable: recognizing complex needs, translating recognition into responsive care, and ensuring the professional and institutional capacity to respond.

The first challenge is recognition. Several contributions demonstrate why vulnerability may be missed when health problems are approached as isolated diseases or outcomes. Yuan et al. documented persistent global inequalities in neonatal encephalopathy, showing how early-life health burdens remain patterned by socioeconomic development and healthcare resources. Importantly, their analysis also points to the role of healthcare professionals' capacity in reducing preventable burden, highlighting multidisciplinary team training, competency in fetal monitoring and resuscitation, and simulation-based training as strategies relevant to prevention and patient safety. At another stage of the life course, Tan et al. demonstrated that symptoms among older adults with type 2 diabetes form interconnected networks rather than isolated clinical manifestations. Identifying core and sentinel symptoms offers opportunities for earlier and more targeted clinical management and greater patient involvement in symptom monitoring. Duan et al. further illustrated the interaction between biological and behavioral pathways by examining the effects of resistance training in older adults with possible sarcopenia, in which changes in the gut microbiota, microbial-derived metabolites, and inflammatory markers accompanied improvements in physical function. Across different scales, these studies show that recognizing vulnerability requires attention to interacting developmental, biological, behavioral, and contextual processes, and, in some settings, professional competencies that enable such risks to be recognized and addressed.

Recognition, however, does not by itself improve care. A second challenge is translating multidimensional understanding into clinical responses. Del Pino-Sedeño et al. evaluated a multicomponent transitional care program for people with complex multimorbidity, illustrating how coordination, individualized support, and continuity of care between hospital and primary care settings can address needs that extend beyond individual diseases. Duan et al. integrated psychological assessment and graded intervention into general hospital care, providing a practical approach to recognizing psychosocial needs alongside physical illness. These studies move the biosocial perspective from description toward action: complexity must not only be identified but incorporated into how care is organized and delivered.

Dube et al. deepen this argument by demonstrating that the translation from recognition to responsive care occurs through relationships between patients and healthcare professionals. Professional nurses providing primary healthcare to LGBTQI communities described knowledge gaps, uncertainty, personal biases, and challenges associated with patients' previous experiences of discrimination. Vulnerability may therefore be intensified when healthcare encounters fail to respond appropriately to patients' lived and social experiences. Together with the professional competency implications identified in the neonatal encephalopathy study, these findings reinforce the relevance of healthcare professions education and continuing professional development, while extending it beyond technical competencies to include culturally responsive communication, critical self-reflection, and inclusive care. More broadly, Dube et al. illustrate that biosocial knowledge becomes clinically meaningful only when professionals possess the capacity to translate it into appropriate practice.

The third challenge concerns that capacity itself. Banjac et al. examined meditative relaxation among hospital physicians, highlighting clinicians' psychological and physiological well-being. Rather than simply recasting healthcare professionals as another vulnerable population, this study raises a different question: whether professionals working within demanding environments have the capacity and support necessary to sustain responsive care. Mirică and Soare extend this issue to the institutional level by examining social insurance physicians in Romania. By linking professional ethics and autonomy to financing structures, resource constraints, and institutional safeguards, their analysis demonstrates that professional practice is enabled or constrained by the systems in which care occurs.

Considered together, the contributions therefore reveal a sequence that is difficult to discern from any individual study: vulnerability must first be recognized in its biological, psychological, and social complexity; that recognition must be translated into responsive clinical practice; and such practice depends on professionals and institutions with sufficient knowledge, well-being, ethical support, and organizational capacity to respond. The integrated biosocial perspective thus functions not simply as a description of interacting determinants but as a framework linking the identification of complex needs to the conditions under which healthcare can address them.

This synthesis also reveals a productive tension. Expanding the ability to recognize vulnerability may improve responsiveness, but labeling individuals or groups as vulnerable can also obscure structural and relational causes or reduce complex experiences to individual characteristics. The LGBTQI healthcare findings particularly remind us that recognition without trust, dignity, cultural responsiveness, and patient agency may itself undermine care. Integrated biosocial approaches must therefore combine greater sensitivity to vulnerability with critical attention to how it is defined and acted upon.

Three priorities follow. First, research should improve the conceptualization and measurement of multidimensional vulnerability while avoiding fixed or stigmatizing labels. Second, intervention research should connect recognition to action by testing multilevel approaches that are responsive to both individual needs and the environments that shape them. Third, implementation research should examine the conditions necessary to sustain these approaches in routine care, including education for healthcare professionals and continuing professional development across technical, psychosocial, cultural, and ethical competencies; interdisciplinary collaboration; organizational support; and ethical safeguards.

The collective lesson of this Research Topic is therefore more specific than a general call for interdisciplinary care. Integrated biosocial medicine requires a continuum from recognition to response to capacity: recognizing vulnerability in its multidimensional forms, translating that recognition into responsive care, and creating professional and institutional conditions that enable such care. This framework offers a practical direction for advancing care for vulnerable populations while placing healthcare professionals and healthcare systems within, rather than outside, the processes through which vulnerability is addressed.

